# Alcohol Binge Drinking Selectively Stimulates Protein S-Glutathionylation in Aorta and Liver of *ApoE*^−/−^ Mice

**DOI:** 10.3389/fcvm.2021.649813

**Published:** 2021-03-16

**Authors:** Kerstin Seidel, Xueping Wan, Mo Zhang, Yuxiang Zhou, Mengwei Zang, Jingyan Han

**Affiliations:** ^1^Vascular Biology Section, Evans Department of Medicine, Whitaker Cardiovascular Institute, Boston University School of Medicine, Boston, MA, United States; ^2^Department of Molecular Medicine, Barshop Institute for Longevity and Aging Studies, Center for Healthy Aging, University of Texas Health Science Center, San Antonio, TX, United States; ^3^Geriatric Research, Education and Clinical Center, South Texas Veterans Health Care System, San Antonio, TX, United States

**Keywords:** *ApoE*^−/−^, cardiovascular disease, aortic endothelial dysfunction, fatty liver, alcohol binge drinking, protein S-glutathionylation, Glutaredoxin-1, Glutathion-S-transferase-Pi

## Abstract

**Background:** Binge drinking has become the most common and deadly pattern of excessive alcohol use in the United States, especially among younger adults. It is closely related to the increased risk of cardiovascular disease. Oxidative stress as a result of ethanol metabolism is the primary pathogenic factor for alcohol-induced end organ injury, but the role of protein S-glutathionylation—a reversible oxidative modification of protein cysteine thiol groups that mediates cellular actions by oxidants—in binge drinking-associated cardiovascular disease has not been explored. The present study defines the effect of alcohol binge drinking on the formation of protein S-glutathionylation in a mouse model of atherosclerosis.

**Methods and Results:** To mimic the weekend binge drinking pattern in humans, ApoE deficient (*ApoE*^−/−^) mice on the Lieber-DeCarli liquid diet received ethanol or isocaloric maltose (as a control) gavages (5 g/kg/day, 2 consecutive days/week) for 6 weeks. The primary alcohol-targeted organs (liver, brain), and cardiovascular system (heart, aorta, lung) of these two groups of the mice were determined by measuring the protein S-glutathionylation levels and its regulatory enzymes including [Glutaredoxin1(Grx1), glutathione reductase (GR), glutathione-S-transferase Pi (GST-π)], as well as by assessing aortic endothelial function and liver lipid levels. Our results showed that binge drinking selectively stimulated protein S-glutathionylation in aorta, liver, and brain, which coincided with altered glutathionylation regulatory enzyme expression that is downregulated Grx1 and upregulated GST-π in aorta, massive upregulation of GST-π in liver, and no changes in Grx1 and GST-π in brain. Functionally, binge drinking induced aortic endothelial cell function, as reflected by increased aortic permeability and reduced flow-mediated vasodilation.

**Conclusions:** This study is the first to provide *in vivo* evidence for differential effects of binge drinking on formation of protein S-glutathionylation and its enzymatic regulation system in major alcohol-target organs and cardiovascular system. The selective induction of protein S-glutathionylation in aorta and liver is associated with aortic endothelial dysfunction and fatty liver, which may be a potential redox mechanism for the increased risk of vascular disease in human binge-drinkers.

## Introduction

Binge drinking is defined by the National Institute on Alcohol Abuse and Alcoholism (1) as a drinking pattern that increase blood alcohol concentration to 0.08 g/dl or higher in a short period of time. It is the most common, costly, and deadly pattern of excessive alcohol use in the United States, particularly among younger adults (2–4). Although numerous epidemiologic studies have revealed a strong positive correlation of binge drinking with cardiovascular disease (e.g., heart attack, atrial fibrillation, atherothrombosis, stroke), the underlying mechanisms are largely unknown (5–8).

Alcohol toxicity is not only directly caused by ethanol but also by its metabolic products, especially the reactive oxygen and nitrogen species (ROS and RNS) (9). These elevated reactive species form an environment favorable to oxidative and nitrosative stress, which have been identified as a unified pathogenesis of cardiovascular disease and tissue damage caused by excessive alcohol consumption (10, 11). The ROS/RNS can readily react with lipids, proteins, and DNA to form cytotoxic oxidation products such as 4-Hydroxynonenal derived from lipid peroxidation, 8-Oxoguanine accumulated in DNA, carboxylation and nitrotyrosine in proteins. So, these oxidation products and modifications have been widely used as markers of oxidative stress (12). These markers are detected in patients with alcoholism as well as animal models of excess alcohol consumption, providing evidence for the involvement of ROS/RNS in pathogenesis of alcohol-induced end organ damage, including cardiomyopathy, liver disease, and neuronal disorders (13–15). Over the past three decades, substantial evidence supports that ROS/RNS can also induce oxidative modifications of cysteinyl thiol groups in target proteins, such as sulfenic acid (Pr-SOH), glutathionylation (Pr-SSG), nitrosylation (Pr-SNO). Of these reversible oxidative modifications, Pr-SSG appears to be the primary one mainly due to the high abundant glutathione (GSH), which catalyze the conversion of -SOH and -SNO into -SSG, or the oxidized GSH (GSSG) after reacting with ROS can directly induce the formation of Pr-SSG. This oxidative modification plays a critical in redox homeostasis and cell biology: (1) it can prevent Pr-SOH from further oxidation, thus acting as a protein protection mechanism against permanent oxidative damage and subsequent degradation; (2) addition of GSH and a net negative charge cause changes in structure and ultimate function of target proteins, thereby mediating ROS/RNS-induced cellular signaling events (16–18). Therefore, Pr-SSG is not only just an indicator of thiol oxidative stress, but also a mechanism of redox signal transduction (19–22). Numerous cardiac and vascular proteins have been identified as targets for S-glutathionylation, indicating its important role in cardiovascular health and disease (19). However, it is unclear whether and/or how binge drinking affects protein S-glutathionylation formation in cardiovascular system and alcohol-targeted organs.

Protein S-glutathionylation is a cyclical process and can be efficiently and specifically removed by glutaredoxin-1 (Grx1) via a thiol-disulfide exchange reaction in the presence of glutathione, NADPH and glutathione reductase (23). Our previous study in *ApoE*^−/−^ mice demonstrated that transgenic overexpression of human Grx1 can reverse metabolic stress-induced aortic protein S-glutathionylation and related barrier dysfunction (24). Our recent studies demonstrated that mTORC1 activation contributes to the effect of chronic ethanol plus binge on lipogenesis and fatty liver in mice and humans (25). S-glutathionylation formation is also catalyzed by glutathione S-transferase family members, which are detoxification enzymes and up-regulated in alcoholic liver disease (26–28). For example, GST-π deficiency leads to decrease in S-glutathionylation following oxidative and nitrosative stress both *in vitro* and *in vivo* (29, 30). However, the impact of binge drinking on this GST-π and Grx1/GR enzymatic regulation system in different tissues has not been defined.

In the present study, we examine the effect of weekend-binge drinking on protein S-glutathionylation and its enzyme regulation system in the alcohol sensitive organs (liver and brain) and cardiovascular system (aorta, heart, lung) of *ApoE*^−/−^ mice—a well characterized mouse model of atherosclerosis. We found that this effect is tissue specific: aorta and liver are particularly susceptive to binge drinking-induced thiol redox regulation, which are accompanied with aortic endothelial dysfunction and fatty liver, providing new insights into cardiovascular effects of alcohol binge drinking.

## Materials and Methods

### Animals

All experimental animal procedures were approved by the Institutional Animal Care and Use Committee (IACUC) at Boston University Medical Campus. ApoE-deficient (*ApoE*^−/−^) mice on a C57BL/6J background were acquired from Jackson Laboratory (#002052, Bar Harbor, ME). 10 to 12- week-old male mice were initially fed the control Lieber-DeCarli diet *ad libitum* for 5 days to acclimate them to a liquid diet and tube feeding. Weekend-binge and control groups were subsequently pair-fed with the isocaloric control diet (Bio-Serv., F1259P). Weekend-binge group received ethanol gavages (5 g/kg of body weight) for two consecutive days every week over the course of 6 weeks; the control group recevied an isocaloric maltose dextrine solution (Bio-Serv, #3653, 9 g maltose dextrine/kg of body weight). During the acclimation phase the food intake was monitored, the average daily amount consumed per mouse was calculated and adjusted for all groups so that both groups consumed equal amounts of diet. During the feeding period, the cages were checked twice (in the morning and afternoon) everyday to ensure that the mouths of the feeding tubes were not clogged or leaking. As the only source of nutrients and water, the liquid diet was changed every day in the afternoon. The dietary intake was recorded daily and bodyweight was record weekly.

### Flow-Mediated Vasodilation Measurement

Flow-mediated vasodilation measurements were performed after 4 weeks of weekend-binge ethanol feeding as described previously (31). Briefly, mice were anesthezized by an intraperitoneal injection of Ketamine (80 mg/kg) and Xylazine (10 mg/kg) and were placed on a heated examination plate to maintain the core body temperature at 37 ± 1°C during the entire FMD measurement. An epilated area of a hindlimb was disinfected and a small incision (~1 mm) in the inner thigh was made to expose the femoral artery. To measure the FMD of the mouse a vascular occluder (Fine Research Tools, #18080-03) was placed around the lower limb to induce occlusion of the femoral artery. Total FMD% value was quantified as the percent change of the diameter 90 s after releasing the cuff as compared to pre-release values: total FMD = [(Diameter^after−release^ – Diameter^pre−release^)/Diameter^pre−release^]×100%.

### Aortic Endothelial Permeability Measurement *in vivo*

For measuring aortic permeability, 0.5% Evans Blue Dye (EBD) dissolved in PBS containing 4% BSA was intravenously administered to the anesthetized mice (30 mg/kg of body weight). Sixty minutes after injection, the animals were perfused with PBS through the left ventricle until colorless perfusion fluid was obtained from the right atrium. The aortic tree was then excised, and en face extravasation of EBD-BSA was photographed.

### Tissue Harvesting and Preparation

Blood was collected via the retro-orbital sinus under anesthesia (ip injection of Ketamine [150 mg/kg] and Xylazine [20 mg/kg]). Plasma was prepared by centrifugation of the collected blood samples at 2,000 × g for 15 min and assayed for cholesterol and triglycerides using the Infinity™ Triglyceride Liquid Stable Reagent (Thermo Fisher Scientific, #TR22421) and Infinity™ Cholesterol Liquid Stable Reagent (Thermo Fisher Scientific, #TR13421). The mice were subsequently euthanized by cervical dislocation and immediately perfused *via* the right ventricle with 30 ml ice-cold phosphate buffered saline (PBS, pH = 7.4) containing 10 mM N-Ethylmaleimide (NEM, Thermo Fisher Scientific, #23030) to alkylate the free protein thiols, and tissue (liver, heart, lung, and brain) and aorta were carefully dissected. Cleared aorta and tissue were flash-frozen in liquid nitrogen or embedded into optimum cutting temperature compound (OCT, Thermo Fisher Scientific, #4583). Serially sections of ascending aortae (10-μm thick) and liver (7-μm thick) were obtained for immunohistochemical analysis.

### Liver Histology

Routine H&E staining of liver frozen sections was performed as described previously (32). Briefly, the sections were fixed with formalin overnight at room temperature and stained with Harris modified hematoxylin/Eosin-Phloxine solution (Newcomer Supply, #1201 and #1082A). The lipid content of liver specimens was analyzed by staining with Oil Red O, and the nuclei were counterstained with hematoxylin. Slides were mounted with VectaMount™ AQ aqueous mounting medium (Vector Laboratories, H-5501) and imaged using upright Olympus microscope with 4x and 20x objectives.

### Immunofluorescence Analysis

For immunofluorescence studies, fresh frozen aorta sections were fixed with ice-cold acetone at −20°C for 10 minutes followed by blocking with 2.5% horse serum for 1 h. The sections were then incubated overnight at 4°C with the primary antibody Grx1 (Abcam, ab187507, 1:500). Primary antibody was detected using the VectaFluor Excel Amplified Anti-rabbit IgG Dylight 594 Antibody Kit (Vector Laboratories, DK-145 1594). For the detection of glutathionylated proteins fresh frozen aorta section were fixed with ice-cold acetone at−20°C for 10 min, followed by incubation with 10 mM N-Ethylmaleimide for 10 min. The sections were then treated with mouse IgG Blocking Reagent (Vector M.O.M. kit, Vector Laboratories. Inc., MKB-2213-1) for 1 h to reduce the background staining caused by endogenous mouse IgG. After blocking with 5% donkey serum for 1 h, sections were incubated overnight at 4°C with anti-GSH (Virogen, #101-A-250, 1:1,000) followed by donkey anti-mouse 594. Fluorescent images were captured on a Nikon deconvolution wide-field epifluorescence microscope equipped with a 20× objective with filter sets for DAPI, FITC, and Texas Red (BU Cellular Imaging Core).

### Immunoblot Analysis

Protein S-glutathionylation and protein content in different tissues were assayed by Western blot analysis as described (24). Briefly, the pulverized tissues were lysed with RIPA buffer (50 mM Tris pH 8.0, 150 mM NaCl, 1% Triton X-100, 0.5% sodium deoxycholate, 1% sodium dodecyl sulfate) supplemented with protease and phosphatase inhibitor cocktail (Thermo Fisher Scientific, A32953 and A32957). The cleared tissue lysates were subjected to Western blot analysis for glutathionylated protein with anti-GSH antibody (Virogen, #101-A-250, 1:500) under non-reducing condition, and other antibodies under reducing conditions: Grx1 (Abcam, ab45953, 1:1,000), GR (abclone, A4566, 1:1,000), GST-π (MLB, 312, 1:1,000) and eNOS (Cell Signaling, 1:1,000). After incubating with secondary antibodies corresponding to their primary antibodies (1: 5,000 dilution), the membranes were developed with the chemiluminescent substrate and imaged using KwikQuant^TM^ Imager system (Kindle Biosciences LLC, D1001). Densitometric quantification was performed using the NIH ImageJ program.

### Quantitative Real-Time PCR

Total RNA was isolated from the tissues using TRIzol^TM^ reagent (Invitrogen, #15596026) according to the manufacturer's instruction, followed by conversion to cDNA using the HiFiScript gDNA Removal cDNA Synthesis Kit (CoWin Biosciences, CW2020). The transcripts were quantified with CFX96 Real-Time PCR System (BioRad, #1855196) by using the UltraSYBR mixture (CoWin Biosciences, CW0957) and ΔΔ*C*_T_ threshold cycle method. Gene expression levels were normalized to those of 18S and presented relative to the control. The specificity of the PCR amplification was verified by melting curve analysis of the final products and primer efficiency test.

The following custom-made primers were used for SYBR Green based qPCR:

18S: F: 5′- ccgccgccatgtctctagt- 3′, R: 5′- gcccatcgatgttggtgttg- 3′;
Grx1: F: 5′- gagaacagttcctcgggtcttc−3′, R: 5′- agctgtgtcagcatggactc -3′;
Grx2: F: 5′- ACTACAAGGCTGTGGAGTTGG−3′, R: 5′- TGGGAACGGTTCTTTCCCCAG -3′;
Gstp1: F: 5′- GCAAATATGTCACCCTCATCTACACC−3′, R: 5′- GCAGGGTCTCAAAAGGCTTCA -3′;
Gstp2: F: 5′- CAAATATGGCACCATGATCTACAGA−3′, R: 5′- GCAGGGTCTCAAAAGGCTTCA -3′

### Statistical Analysis

Data were presented as mean ± standard error (S.E.M). Statistical analysis was performed using Prism 9.0 (GraphPad Software). Means were compared between two groups by the Mann-Whitney *U* test. Multiple comparisons were conducted with 1-way ANOVA followed by Dunnett test. A value of *p* < 0.05 was considered statistically significant.

## Results

### Ethanol-Binge Drinking Increases the Protein S-Glutathionylation in the Aorta, Liver, and Brain of *ApoE*^–/–^ Mice

**Figure 1:**
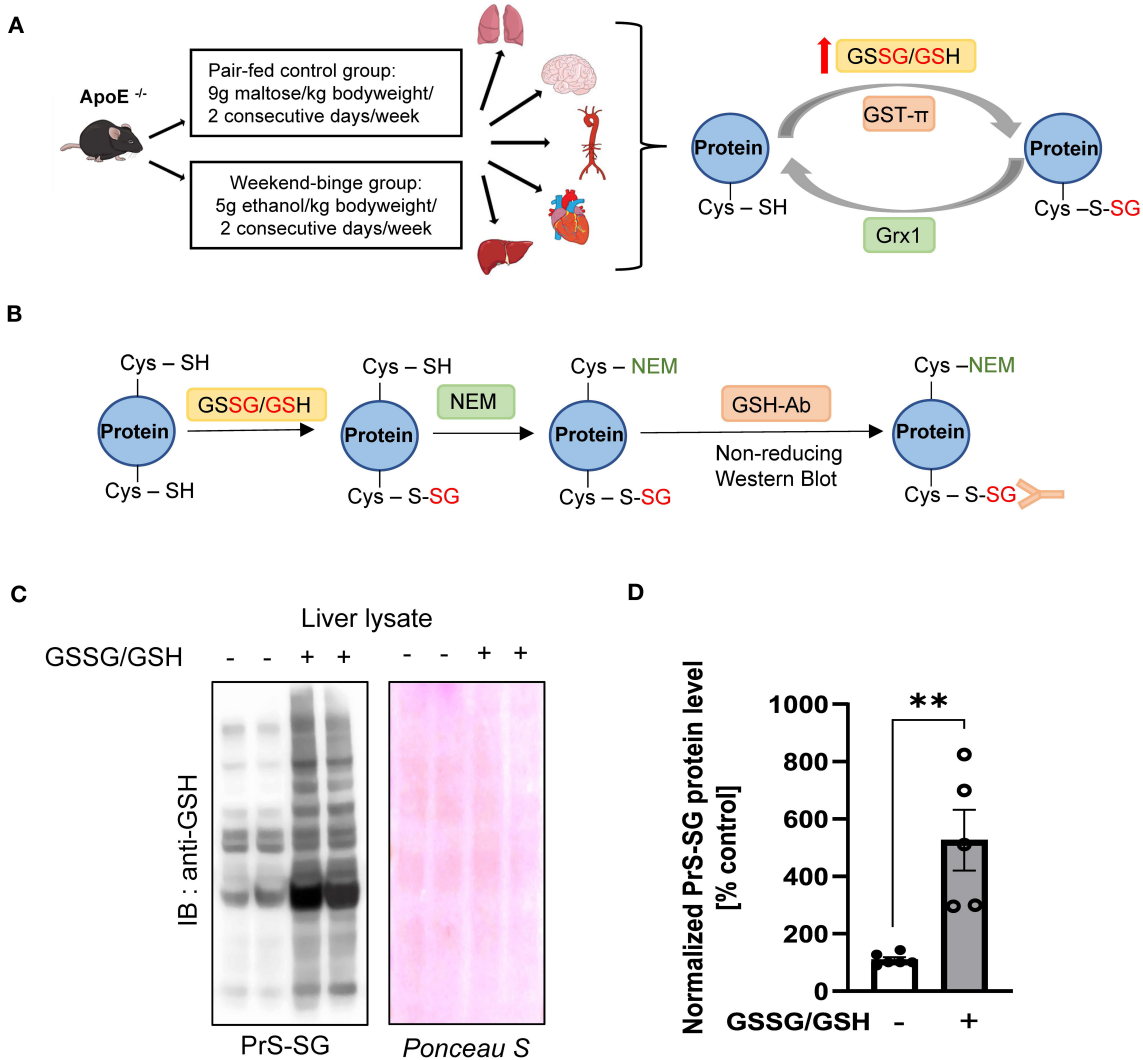
Schematic overview of experimental setup and Validation of the GSH antibody. **(A)** Schematic detailing the experimental groups and analyzed tissue and simplified schematic representation of the Protein S-glutathionylation and De-glutathionylation cycle. *ApoE*^−/−^ mice were fed the control Lieber-DeCarli diet ad libitum for 6 weeks and Weekend-binge (WB) group received ethanol gavages (5 g/kg of body weight) for two consecutive days every week over the course of 6 weeks and the control group an isocaloric maltose dextrine solution (9 g/kg of bodyweight) respectively. After 6 weeks the mice were sacrificed and liver, heart, aorta, brain, and lung tissue harvested and analyzed for Protein S-glutathionylation (PrS-SG), Glutaredoxin1 (Grx1), and Glutathion – S – transferase π (GST-π) expression profile. **(B–D)** Validation of the performance of the anti-GSH antibody. **(B)** Schematic representation of the experimental workflow. Liver lysate was incubated without or with GSSG/GSH on ice for 30 min and afterwards free Cysteine residues alkylated with N-Ethylmaleimide (NEM). After removing unbound GSSG/GSH/NEM the samples were subjected to 4–12% NuPAGE Bis-Tris SDS-PAGE under non-reducing conditions and immunoblot was performed with GSH antibody. **(C)** Representative immunoblot result and Ponceau protein staining. **(D)** Densitometric analysis of PrS-SG signal intensity in the liver lysate of GSSG/GSH treated samples. Bars represent mean ratio ± error propagated S.E.M. (*n* = 5, Student *t* test, **p-value < 0.01).

As depicted in Figure 1A, *ApoE*^−/−^ mice were fed weekend-binge ethanol (5 g/kg body weight/day, 2 consecutive days/week) or pair-fed control (isocaloric maltose dextrin/day, 2 consecutive days/week) for 6 weeks. Body weight and food intake of pair-fed control and weekend-binge ethanol *ApoE*^−/−^ mice are summarized in Table 1. Aorta, heart, lung, brain, and liver were then collected and subjected to biochemical analysis for protein S-glutathionylation and its regulatory system. Prior to accessing the protein S-glutathionylation levels in these alcohol-sensitive organs of *ApoE*^−/−^ mice, we experimentally reaffirmed the specificity of the anti-GSH antibody in the recognition of S-glutathionylated proteins under non-reducing conditions. As shown in Figures 1B,C, *in vitro* incubation of liver homogenates with a mixture of GSSG and GSH induced ~6-fold formation of S-glutathionylation, which was detected by non-reducing Western blot analysis with anti-GSH antibody.

**Table 1:** Overview of bodyweight and food intake in *ApoE*^−/−^ mice.

**Group**	**Body weight (g)**	**Daily food intake (ml)/mouse**
Pair-fed control group (CTL)	26.21 ± 1.49	9.89 ± 0.31
Weekend-binge drinking group (WB)	30.43 ± 1.32	9.64 ± 0.66

**Figure 2:**
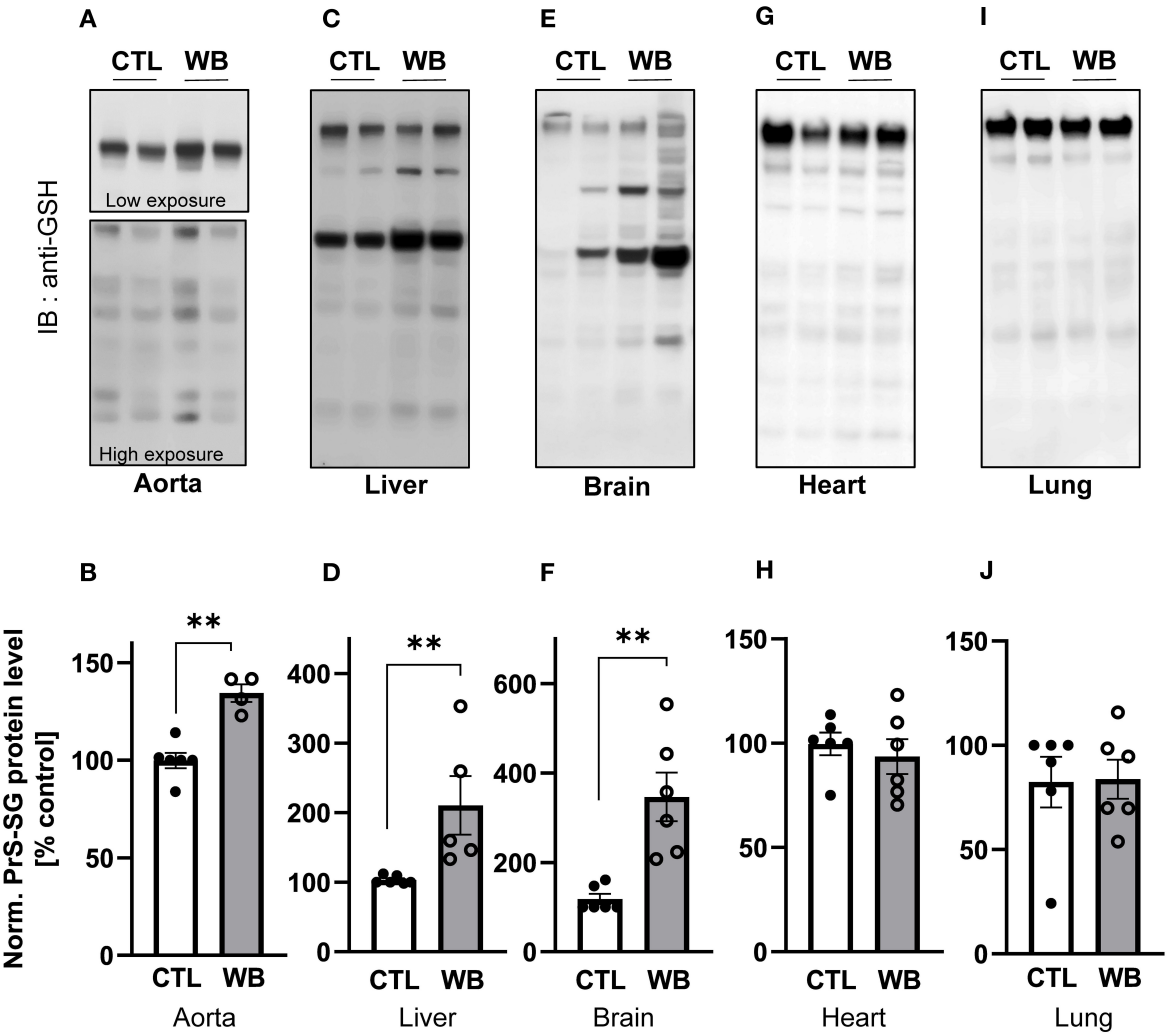
Alcohol-binge drinking increases the protein S-glutathionylation in aorta, liver, and brain of *ApoE*^−/−^ mice Protein S-glutathionylation (PrS-SG) level from *ApoE*^−/−^ mice in **(A,B)** aorta; **(C,D)** liver; **(E,F)** brain; **(G,H)** heart and **(I,J)** lung tissue homogenate of control (CTL) and weekend-binge (WB) ethanol-fed mice were analyzed. Protein of different tissues were isolated and immunoblot under non-reducing conditions was performed. PrS-SG levels were detected with the anti-GSH antibody. **(A,C,E,G,I)** Representative immunoblot results and **(B,D,F,H,J)** densitometric analysis of PrS-SG signal intensity in the tissue lysate. Bars represent mean ratio ± error propagated S.E.M. (*n* = 4–6, Student *t* test, **p < 0.01).

We next investigate the influence of ethanol binge-drinking on protein S-glutathionylation in aorta, heart, lung, brain, and liver of *ApoE*^−/−^ mice (Figure 2). We found that compared with control, ethanol binge drinking significantly stimulated formation of S-glutathionylation by 1.3-fold in aorta (Figures 2A,B), by 2-fold in liver (Figures 2C,D), and by 2-fold in brain (Figures 2E,F), while no significant differences were observed in heart (Figures 2G,H) or lung (Figures 2I,J). Collectively, these results indicated that ethanol binge drinking selectively increased protein S-glutathionylation in aorta, liver and brain in a mouse model of atherosclerosis.

### Ethanol-Binge Drinking Stimulates Aortic Protein S-Glutathionylation Formation in the Aorta of *ApoE*^–/–^ Mice Likely Through, Diminished Grx1 Protein and Upregulated GST Levels

**Figure 3:**
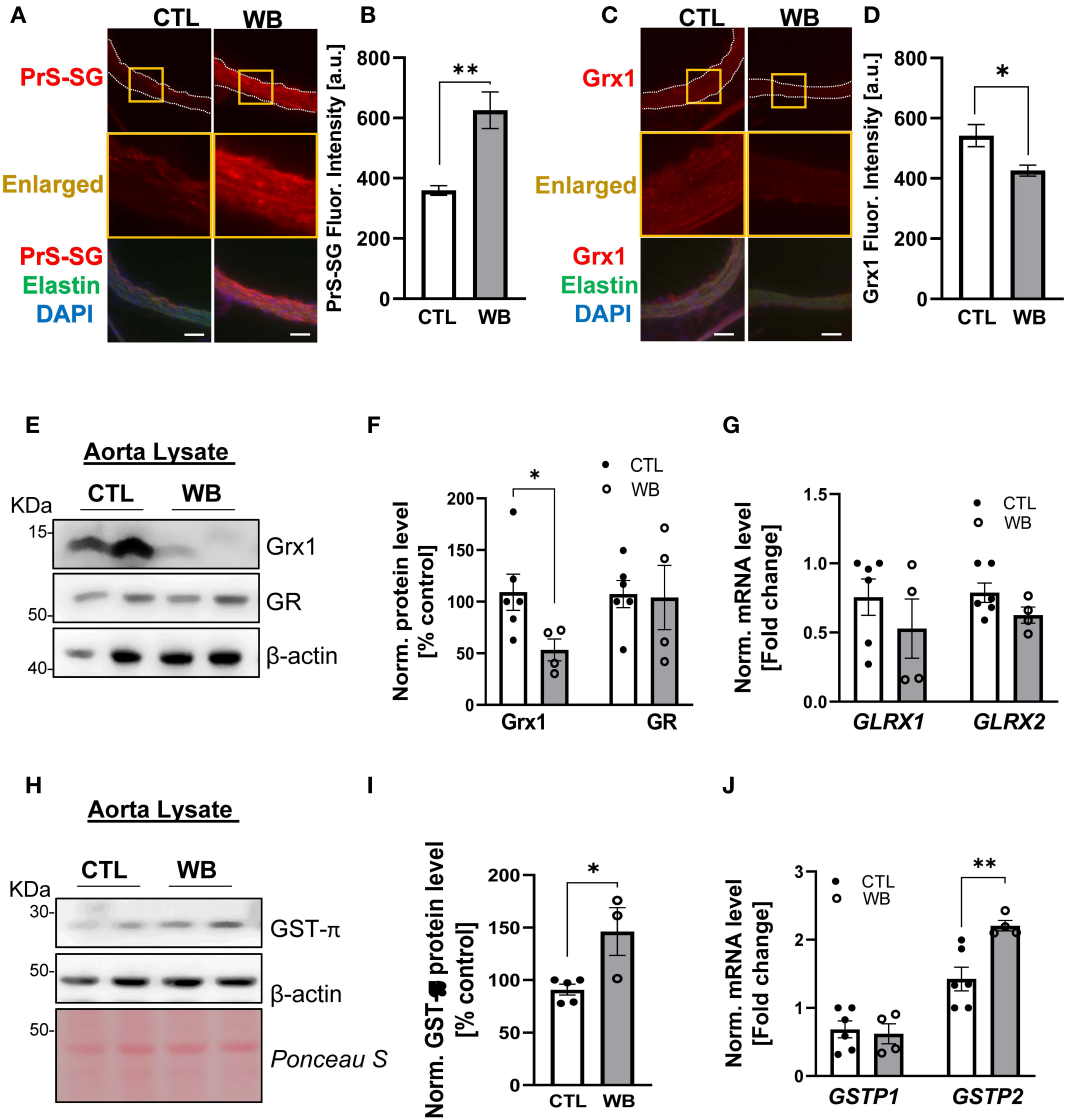
Weekend-binge drinking leads to decreased Grx1 and increased GST-π expression in aortic tissue in *ApoE*^−/−^ mice. **(A–D)** Immunofluorescence analysis of **(A,B)** PrS-SG and **(C,D)** Grx1 expression in the aorta of pair-fed control (CTL) or Weekend-binge ethanol-fed (WB) *ApoE*^−/−^ mice. Fresh and frozen cross-sections of aorta without evident lesions were immunostained for **(A)** PrS-SG and **(C)** Grx1 and fluorescence (red) was then **(B,D)** quantified using Icy ImageJ software. Nuclei were counterstained with DAPI (blue), and elastin was visualized via its autofluorescence (green). The vessels are outlined in white dashed lines, yellow boxes indicate the area shown in higher magnification below the corresponding images. For each aortic cross-section the red fluorescence intensity was measured in three randomly selected image fields, average, and expressed as arbitrary units (A.U.). **(E–J)** Descending aorta from *ApoE*^−/−^ mice from control (CTL) and Weekend-binge (WB) ethanol-fed mice were analyzed for **(E–G)** Grx1, GR, and **(H–J)** GST-π using immunoblotting and qPCR. Weekend-binge ethanol feeding reduces the expression of **(E,F)** Grx1 on protein but not **(G)** mRNA level in *ApoE*^−/−^ mice compared to control group and has no effect **(E–G)** on the expression of GR. **(H–J)** In the weekend-binge group the level of GST-π **(H,I)** protein and **(J)** mRNA is higher than those in the controls. **(E,H)** Representative immunoblot results and **(F,I)** densitometric analysis of Grx1, GR, and GST-π in the tissue lysate. **(G,J)** mRNA results of *GLRX1, GLRX2, GSTP1*, and *GSTP2* in the tissue lysate. Bars represent mean ratio ± error propagated S.E.M. (*n* = 4–6, Student *t* test, **p < 0.01, *p < 0.05).

Immunofluorescence analysis of aortic cross-sections reaffirmed that arterial protein S-glutathionylation was stimulated by ethanol binge drinking. The immunofluorescence signal intensity of glutathionylated proteins was significantly increased by 1.5-fold in aortae from binge drinking group compared with control group (Figures 3A,B). Next, we analyzed the mRNA and protein levels of GST-π, Grx1 and GR, the key S-glutathionylation regulatory enzymes, in aortae of two groups of mice, (Figure 1A). Compared with the control group, the protein levels of Grx1 were significantly decreased in aortae of the binge drinking group, as measured by immunofluorescence analysis of aortic cross sections (25% of control group, Figures 3C,D) and confirmed by Western blot analysis (50% of control group, Figures 3E,F). Interestingly, the mRNA level was not significantly altered by binge drinking, implying that these proteins are post-translationally degraded (Figure 3G). As a Grx1 regenerating enzyme, GR content was not affected by binge drinking. In contrast, both mRNA and protein levels of GST-π were increased 2-fold in aortae of binge drinking group mice (Figures 3H–J). Taken together, these data indicate that binge drinking stimulates aortic protein S-glutathionylation, which is possibly attributed to dysregulation of the glutathionylation/deglutathionylation enzyme levels.

### Ethanol-Binge Drinking Causes Impaired Aortic Endothelial Function of *ApoE*^–/–^ Mice

**Figure 4:**
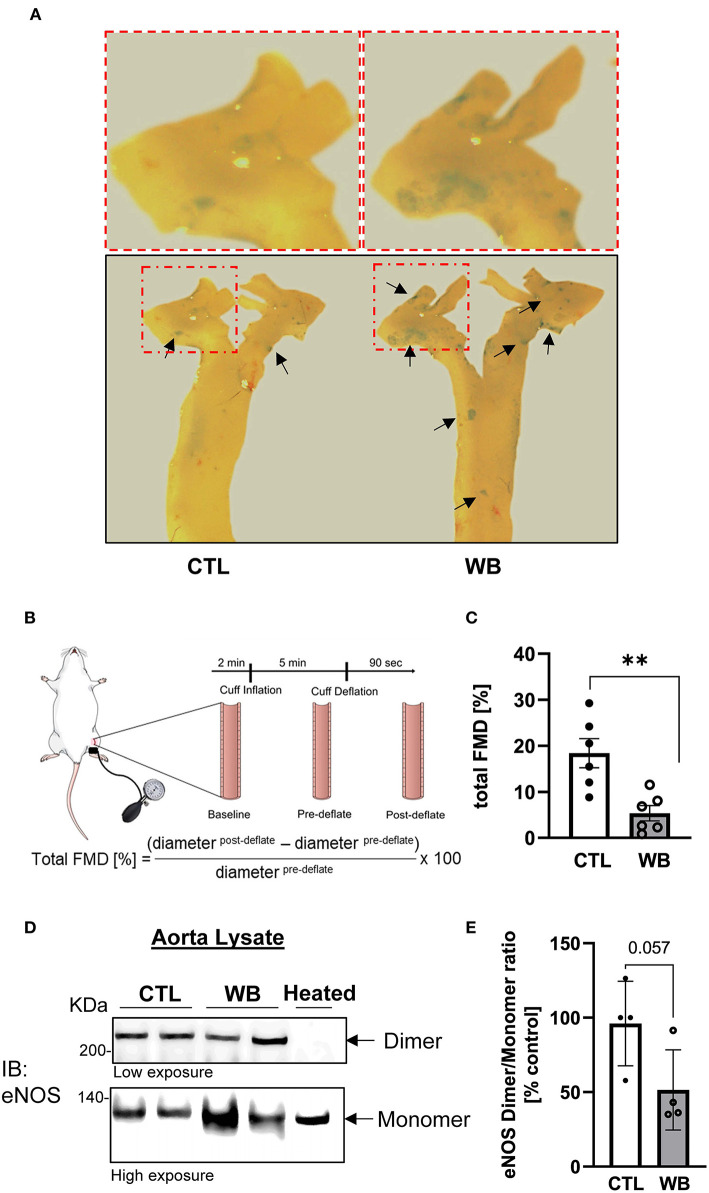
Impaired vascular function and hyperpermeability in the aorta of weekend-binge ethanol-fed *ApoE*^−/−^ mice. **(A)** Ethanol-induced increase in aortic permeability. Aortic permeability in control and ethanol-fed *ApoE*^−/−^ mice was assessed by BSA-Evans blue dye (BSA-EBD) conjugate permeation method. Representative en face microscopy image of aortic arch segment from control and ethanol-fed mice. Area of hyperpermeability is marked by black arrows, red marked boxes indicate the area shown in higher magnification above the images. **(B)** Schematic representation of *in vivo* imaging of a mouse femoral artery for measurement of flow-mediated vasodilation (FMD). Epilated area of hindlimb is disinfected and a small incision (~1 mm) in the inner thigh is made to expose femoral artery. To measure the FMD of the mouse a vascular occluder is placed around the lower limb to induce occlusion of the femoral artery for 5 min. Vessel diameter is measured pre- and post deflation of the cuff and the total FMD calculated. **(C)** Ethanol-induced decrease in total FMD in weekend-binge (WB) *ApoE*^−/−^ mice compared to controls as indicator of an impaired vascular function. **(D,E)** In the weekend-binge group the eNOS Dimer/Monomer ratio is decreased. A heated sample was used as an eNOS monomer control. **(D)** Representative immunoblot result and **(E)** Densitometric analysis of eNOS Dimer/Monomer signal intensity ration in aorta lysate. Bars represent mean ratio ± error propagated S.E.M. (*n* = 4–6, Student *t*-test, **p < 0.01).

In *ApoE*^−/−^ mice, the induction of aortic protein S-glutathionylation is demonstrated to contribute to hypercholesterolemia-induced aortic endothelial hyperpermeability, which is a hallmark of vascular dysfunction and protected by the increase in Grx1 (24). Therefore, we would test the hypothesis that ethanol-binge drinking-induced protein S-glutathionylation causes aortic endothelial dysfunction in *ApoE*^−/−^ mice (Figure 4). First, our results showed that the integrity of aortic endothelial barrier in binge-drinking group was disrupted, which was reflected by the increased permeation of albumin-Evans blue dye (EBD) complex in the aortic arch area—the atherosclerosis prone area of aorta (as shown in the representative photomicrograph of Figure 4A). Secondly, it has been well established that flow-mediated vasodilation (FMD) represents the extent of endothelial activation in response to shear stress, thus serving as a gold standard for evaluating vascular endothelial function in humans and predicting the outcome of cardiovascular disease in the clinical setting (33). We thus used an optical coherence tomography-based angiography imaging technique (31) to measure FMD of femoral artery and to assess the effect of binge-drinking on arterial endothelial function, which was schematically depicted in Figure 4B. In binge-drinking group, the FMD was significantly reduced by 72% compared with control group, indicating a detrimental effect of binge-drinking on aortic endothelial function (Figure 4C). It has been established that eNOS dimerization is necessary for normal eNOS function (34) and regulation of vascular tone. We therefore analyzed the eNOS Dimer/Monomer ration using a low-temperature SDS-PAGE under non-reducing conditions. We observed a decreasing trend in the Dimer-Monomer ration (Figure 4D) in aortic tissue of the binge-drinking *ApoE*^−/−^ mice compared with control mice. In addition, we evaluated the effect of binge-drinking for 6 weeks on atherosclerotic lesion development in *ApoE*^−/−^ mice. There was no significant increase in the lesion area of the aortic root in binge drinking group (data not shown). Taken together, these results clearly suggest that weekend binge drinking can impair vascular endothelial function, which is known to precede the development of atherosclerosis.

### Ethanol-Binge Drinking Up-Regulates GST-π Expression in the Liver of *ApoE*^–/–^ Mice, Which Is Accompanied by Lipid Accumulation

**Figure 5:**
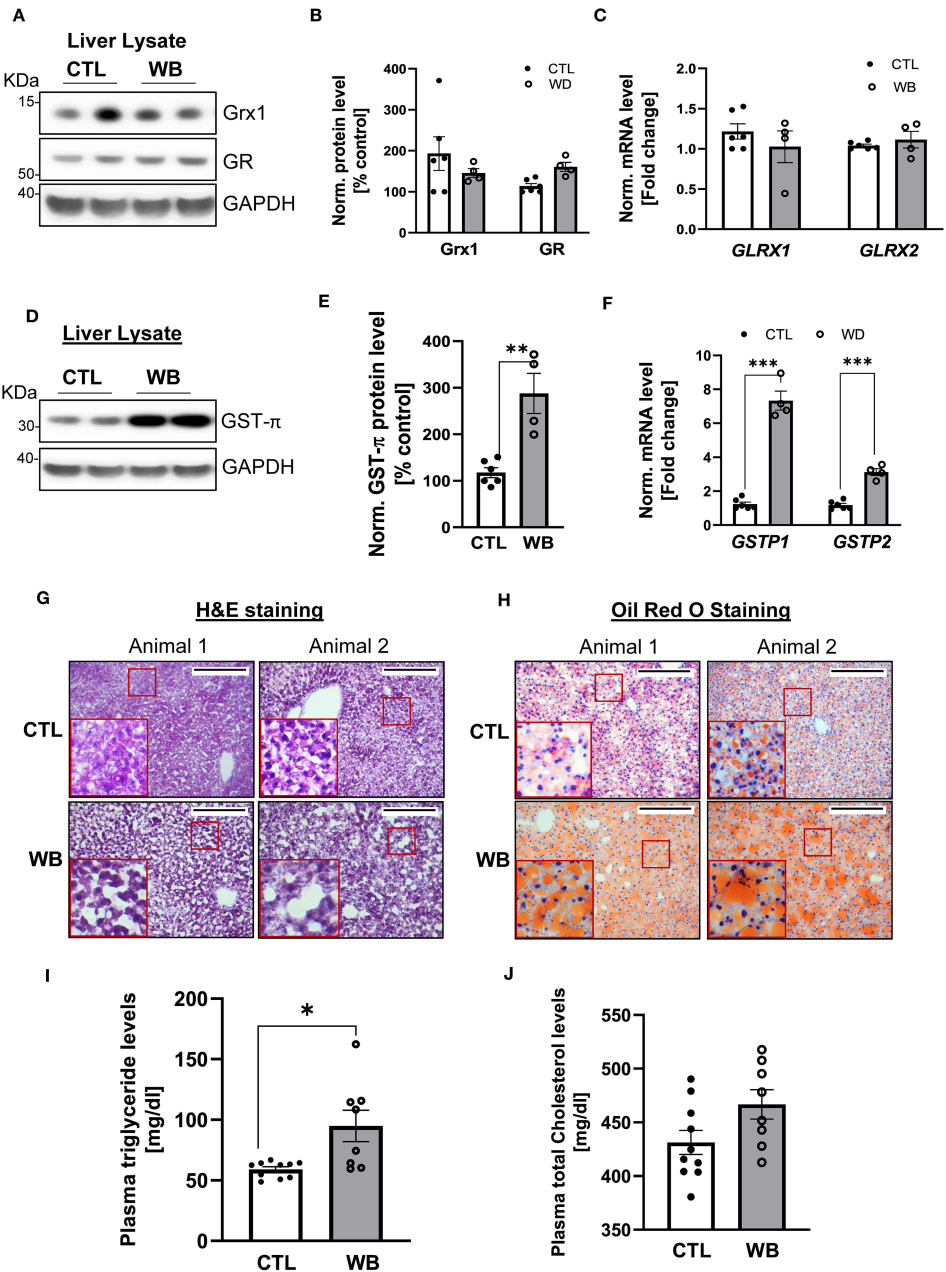
In liver tissue, GST-π expression and lipid accumulation is increased in weekend-binge ethanol-fed ApoE^−/−^ mice compared with the control group. Liver tissue homogenate from control (CTL) and weekend-binge (WB) ethanol-fed mice were assessed for the expression of Grx1, GR and GST-π. **(A–C)** Grx1 and GR level were not significantly altered by ethanol feeding compared with the control group **(A,B)** either on protein nor **(C)** mRNA expression level. Only GST-π was markedly increased **(D,E)** on protein and **(F)** mRNA level. **(G,H)** Liver histology of control (CTL) and weekend-binge (WB) ethanol-fed ApoE^−/−^ mice are performed on 10 μm frozen sections and shown in **(G)** Hematoxylin-eosin staining and **(H)** Oil Red O staining a stronger accumulation of lipids in the weekend-binge group compared to the control group. Red boxes indicate the area shown in higher magnification. Scale bars = 100 μm. **(I)** Significant increase in plasma triglycerides in ethanol-fed ApoE^−/−^ mice (WB) compared to control (CTL) group but **(J)** no increase in total cholesterol plasma level. **(A,D)** Representative immunoblot results and **(B,E)** densitometric analysis of Grx1, GR and GST-π in the tissue lysate. **(C,F)** mRNA results of *GLRX1, GLRX2, GSTP1* and *GSTP2* in the tissue lysate. Bars represent mean ratio ± error propagated S.E.M. (*n* = 4–6, Student *t* test, ***p < 0.001, **p < 0.01, *p < 0.05).

The liver is the main organ that metabolizes alcohol and is therefore the primary target for alcohol-induced toxicity (35). We observed that binge drinking stimulated the formation of protein S-glutathionylation in the liver of *ApoE*^−/−^ mice (Figures 2C,D). To further define the effect of binge drinking on the glutathionylation/de-glutathionylation enzyme regulatory system in the liver, we analyzed the mRNA and protein expression level of GST-π, Grx1 and GR. Liver tissue from alcohol binge-drinking mice displayed a 2.5-fold increase of GST-π: protein levels (Figures 5D,E) and mRNA levels which increased by three to seven-fold (Figure 5F). However, ethanol binge drinking did not significantly change the protein or mRNA level of Grx1 and GR (Figures 5A–C). To test whether ethanol binge drinking can cause fatty liver in a mouse model of atherosclerosis, the most common liver disease associated with alcohol abuse (36), we performed H&E and Oil Red O staining on liver sections. The histological analysis indicated that the lipid accumulation was increased in livers of ethanol binge-drinking animals, which was reflected by the increased paranuclear hepatocellular vacuoles in the H&E-stained sections and more intensively stained neutral triglycerides and lipids in the Oil Red O stained sections (Figures 5G,H). The effect of binge drinking on circulating lipids was also assessed. The plasma level of triglyceride was significantly higher (60% higher compared with control group, Figure 5I) whereas cholesterol levels in binge drinking group showed an increasing trend, but the value did not reach significance compared with the control group (Figure 5J). Collectively, these data indicate that weekend-binge drinking for 6 weeks can greatly stimulate the expression of GST-π and the formation of S-glutathionylation in livers of *ApoE*^−/−^ mice, which coincided with the development of fatty liver.

### Ethanol-Binge Drinking Induced the Formation of Protein S-Glutathionylation in Brain Tissues Without Altering GST-π/Grx1 Regulatory Enzyme System

**Figure 6:**
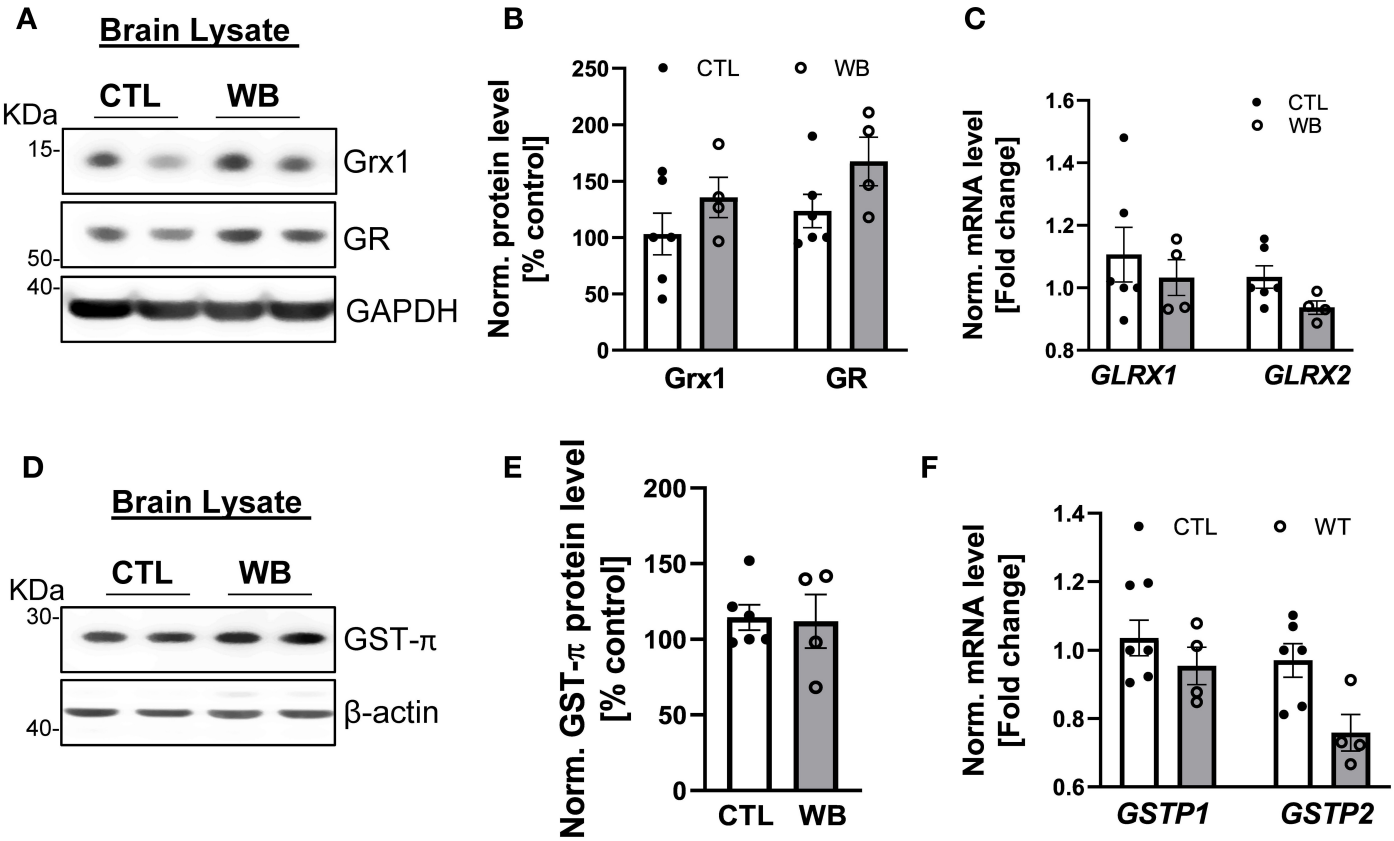
Weekend-binge drinking has no effect on the expression of Grx1, GR and GST-π in brain tissue despite increased protein S-glutathionylation. **(A–F)** Brain tissue of control (CTL) and Weekend-binge (WB) ethanol-fed mice show no alteration in the expression of Grx1 and GR **(A,B)** on protein or **(C)** mRNA expression level. Additionally, no significant changes in the **(D,E)** protein expression and **(F)** mRNA expression of GST-π were observed. **(A,D)** Representative immunoblot results and **(B,E)** densitometric analysis of Grx1, GR and GST-π in the tissue lysate. **(C,F)** mRNA results of *GLRX1, GLRX2, GSTP1* and *GSTP2* in the tissue lysate. Bars represent mean ratio ± error propagated S.E.M. (*n* = 4–6, Student *t* test).

Although binge drinking can significantly induce the formation of protein S-glutathionylation in the brains of *ApoE*^−/−^ mice (Figures 2E,F), there was no significant difference in the protein and mRNA expression level of Grx1, GR (Figures 6A–C) or GST-π (Figures 6D–F) in the brains from binge drinking and control animals. These data suggest that binge drinking-induced protein S-glutathionylation in the brain is not mainly attributed to dysregulation of the Grx1/GST centered glutathionylation regulatory system. In alcohol-dependent patients, oxidized GSH (GSSG) in the brain tissue was markedly increased (37) and it is known that GSSG can directly catalyze protein S-glutathionylation via a thiol disulfide exchange reaction with protein thiol groups. Therefore, it is conceivable that herein, the increased protein S-glutathionylation might be due to the GSSG increase in brain tissues of *ApoE*^−/−^ mice after 6-week ethanol binge drinking.

### Ethanol Binge-Drinking Showed No Effect on Protein S-Glutathionylation and Grx1/GST-π Regulatory System in Heart and Lung of *ApoE*^–/–^ Mice

**Figure 7:**
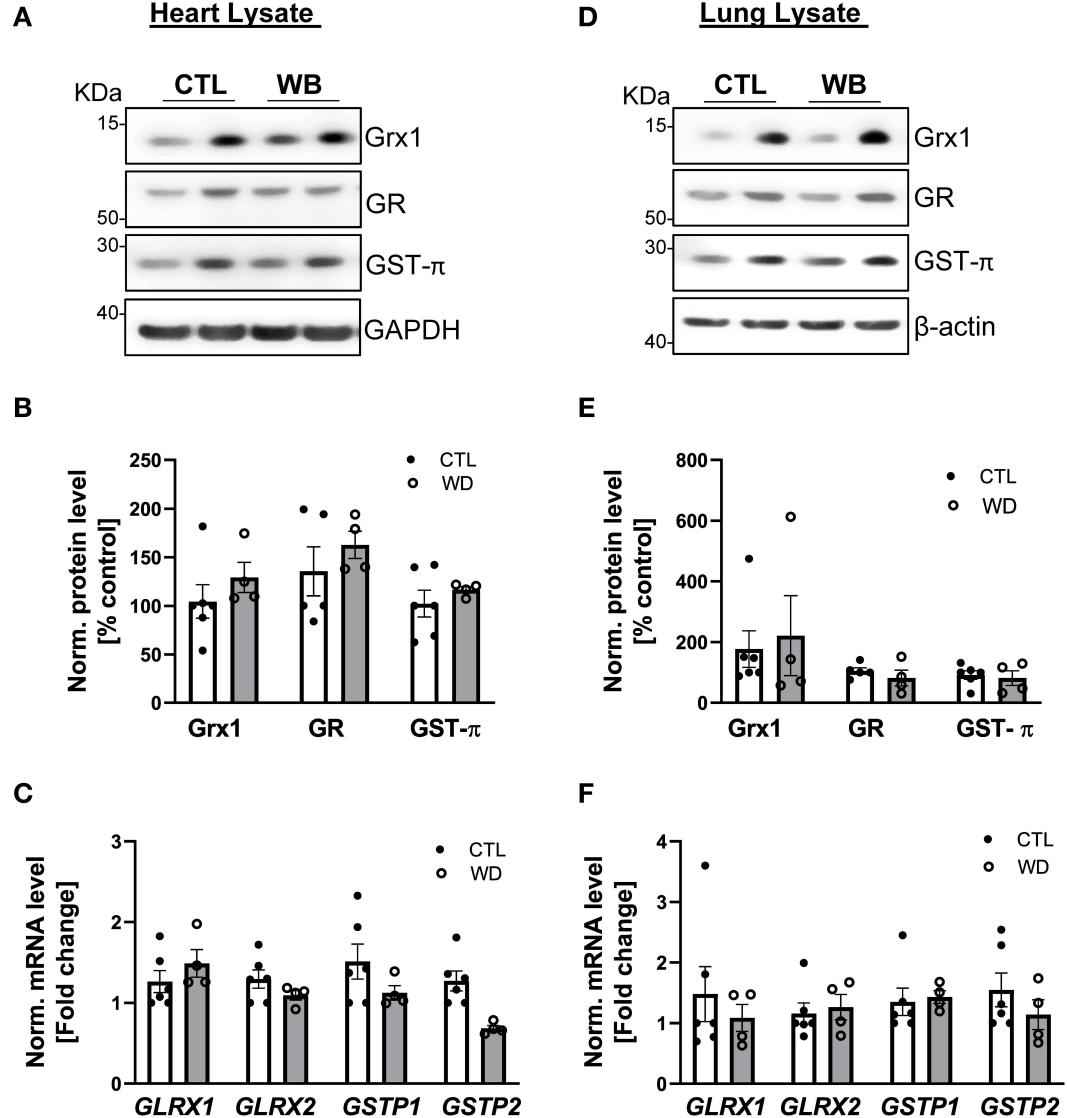
In heart and lung tissue, Grx1, GR and GST-π levels were not significantly altered in weekend-binge ethanol-fed *ApoE*^−/−^ mice compared with the control group. **(A–C)** Heart and **(D–F)** lung tissue homogenate from control (CTL) and weekend-binge (WB) ethanol-fed mice were assessed for the expression of Grx1, GR and GST-π. **(A,B)** Grx1, GR and GST-π protein level and **(C)** mRNA level of *GLRX1, GLRX2, GSTP1* and *GSTP2* was not altered in the heart tissue of the weekend-binge (WB) group as well as in the **(D–F)** lung tissue. **(A,D)** Representative immunoblot results and **(B,E)** densitometric analysis of Grx1, GR and GST-π in the tissue lysate. **(C,F)** mRNA results of *GLRX1, GLRX2, GSTP1* and *GSTP2* in the tissue lysate. Bars represent mean ratio ± error propagated S.E.M. (*n* = 4–6, Student *t* test).

Heart and lung are the primary target organs for the detrimental effects of alcohol abuse (6, 38). However, in these two organs of *ApoE*^−/−^ mice, ethanol binge drinking for 6 weeks did not cause significant changes in proteins S-glutathionylation (Figures 2G–J), or in the protein and mRNA expression levels of Grx1, GR and GST-π (heart: Figures 7A–C, lung: Figures 7D–F). Collectively, these results clearly indicate that ethanol binge drinking affects protein S-glutathionylation and related regulatory enzyme system in a tissue specific manner. This could be explained by two scenarios: (1) there may be powerful antioxidant systems in lung and heart tissues (e.g., superoxide dismutase and catalase) to maintain ROS homeostasis after ethanol binge exposure. Accordingly, protein S-glutathionylation as a ROS-mediated event remains constant; (2) The Grx-centered deglutathionylation system is robust and able to timely remove the formed S-glutathionylation, so the levels remain unchanged.

## Discussion

Our present study demonstrates that ethanol binge drinking, a risk factor for CVD, can selectively increase protein S-glutathionylation in the aorta, liver and brain of *ApoE*^−/−^ mouse model of atherosclerosis. This increase in glutathionylated proteins coincides with the significant upregulation of GST-π observed in the liver, or concurrently with the GST-π upregulation and the Grx1 downregulation in the aorta. Furthermore, we showed that weekend-binge drinking is associated with aortic endothelial dysfunction and increased liver lipid accumulation. This work provides the first *in vivo* evidence for vascular effect of ethanol binge drinking from the perspective of thiol redox regulation. In view of the important role of protein S-glutathionylation in the pathogenesis of vascular dysfunction this work will provide new insights into S-glutathionylation-centered redox mechanism for binge drinking-induced cardiovascular pathologies.

In this study, we used the *ApoE*^−/−^ mouse model of atherosclerosis to study the cardiovascular effects of binge drinking. The reasons for choosing this animal model are as follows: (1) *ApoE*^−/−^ mice are well-recognized as a best characterized and most widely used animal model for CVD research. Compared with its background strain C57BL/6J, the plasma levels of total cholesterol are increased 4–5-fold. Furthermore, *ApoE*^−/−^ mice develop a spectrum of lesions and proatherogenic events such as vascular endothelial dysfunctions, which are similar to humans (39, 40). (2) Importantly, Liu et al. reported that in *ApoE*^−/−^ mice, weekend-binge drinking for 4 weeks accelerated the progression of carotid artery lesions caused by partial carotid ligation (5). In our study, we did not include atherogenic diet interventions and surgery procedures to promote lesion development, because the major goal of this study was to determine the role of the thiol oxidative modification and its regulatory system in alcohol-sensitive organs caused by binge drinking itself. However, in our study, 6 weeks of binge drinking did not show significant effect on lesion development (data not shown); this negative result might have been due to the short period of ethanol treatment, which is considered a limitation of our study.

The main finding in this study was that ethanol binge drinking selectively increase protein S-glutathionylation in aorta, liver and brain tissue but not in the lung and heart of *ApoE*^−/−^ mice. This tissue specific effect of binge drinking was not expected, because S-glutathionylation is generally associated with increased oxidative/nitrosative stress, which is known to be induced in heart and lung tissues by excessive alcohol ingestion (38, 41). We found that levels of Grx1/GR and GST-π in these two organs were not affected by binge drinking, which may account for S-glutathionylation homeostasis even under oxidative stress conditions. In addition, we found that binge drinking differentially affected expression of Grx1/GR and GST-π in aorta, liver and brain. The protein S-glutathionylation was likely promoted: (1) in aorta via concurrent GST-π upregulation and Grx1 downregulation; (2) in liver *via* massive upregulation of GST-π; and (3) in brain *via* other mechanisms than Grx1/GR and GST-π system. Further research is warranted to elucidate whether and how Grx1 and GST-π mediate binge drinking-induced protein S-glutathionylation, providing new therapeutic strategies targeting S-glutathionylation-centered pathologies that underlie binge drinking-induced damage in the cardiovascular system and liver.

Another major finding of this study was that, ethanol binge drinking can cause fatty liver and vascular endothelial dysfunction, which are known to promote atherosclerosis (42–44). Our previous studies indicate that induction of protein S-glutathionylation is causatively linked to development of fatty liver and vascular endothelial dysfunction, which could be protected and reversed by Grx1 (24, 32). Current work provides a possible mechanistic connection between the induction of protein S-glutathionylation and these proatherogenic events caused by binge drinking. So, it is intriguing to test this hypothesis that would provide new redox mechanisms contributing to pathogenesis of CVD associated with binge drinking.

Protein S-glutathionylation is an oxidant-induced reversible posttranslational modification of protein cysteine residues. It has been historically viewed as a cellular defense mechanism that prevents irreversible oxidation of cysteinyl thiol groups, thereby protecting target proteins from permanent oxidative damage and degradation (20). Recently, increasing evidence support that the addition of GSH and a net negative charge can cause changes in the structure and function of target proteins, thus mediating oxidative signaling events (19). Depending on the context of pathophysiological conditions as well as target proteins, S-glutathionylation may mitigate or mediate organ dysfunction caused by oxidative stress. The present study is sought to characterize the impact of ethanol binge drinking on protein S-glutathionylation in cardiovascular system of mouse model of atherosclerosis, because this oxidative modification is emerging as an important redox mechanism for both cardiovascular health and disease. There are numerous proteins in heart and vessels that are susceptible to redox regulation via S-glutathionylation. For instance, compelling *in vitro* evidence demonstrate that glutathionylation of endothelial NO-synthetase (eNOS) can cause its “uncoupling,” a process that switch eNOS enzymatic activity to generate superoxide rather than NO. This impaired eNOS activity is causatively related to vascular dysfunction (45, 46). Our previous study demonstrated that glutathionylation of Rac1, a small RhoGTPase is linked to aortic vascular endothelial hyperpermeability caused by metabolic stress *in vivo* (24). In addition, our previous studies (32, 47) show that high- fat diet and Grx1 gene deletion can promote protein S-glutathionylation in mouse liver, which is related to non-alcoholic fatty liver and dyslipidemia, and S-glutathionylation of sirtuin-1 is identified as an underlying redox mechanism. Although herein Grx1 remains constant in the liver of binge drinking mouse group, GST-π was significantly upregulated, which is known to catalyze protein S-glutathionylation after oxidative and nitrosative stress (29). Accordingly, we speculate that binge drinking-associated increase in hepatic protein S-glutathionylation may be catalyzed by the upregulated GST-π, and contribute to the development of fatty liver and elevated plasma triglyceride levels. Increased protein S-glutathionylation is also implicated in pathophysiology of lung diseases, such as idiopathic pulmonary fibrosis, asthma, and chronic obstructive pulmonary disease (48). Grx1 deficient mice were more susceptible to bleomycin-induced lung fibrosis, which is accompanied with an increased overall protein S-glutathionylation; the glutathionylated Fas receptor and subsequent activation of the cell death pathway appear to be an important pathogenic mechanism (49). Similarly, protein S-glutathionylation emerged as a redox mechanism involved in the development of neurodegenerative diseases, for example, Alzheimer disease, Parkinson disease and Huntington disease (50).

In summary, this study demonstrated that weekend binge drinking can cause vascular endothelial dysfunction and fatty liver in *ApoE*^−/−^ mice, in a mouse model of atherosclerosis. Importantly, these detrimental effects of binge drinking are accompanied by the selective induction of protein S-glutathionylation—a reversible oxidative modification mediating cellular responses to oxidative/nitrosative stress—in the aorta and liver, in which two key S-glutathionylation regulatory enzymes Grx1 and GST-π are also differentially modulated by binge drinking. This work is the first attempt to establish a connection between the vascular effect of binge drinking with dysregulation of thiol redox homeostasis, providing a fertile ground for future research on the role of protein S-glutathionylation in cardiovascular pathologies associated with alcohol binge drinking.

## Data Availability Statement

The data underlying this article are available in the article and in its online supplementary material.

## Ethics Statement

The animal study was reviewed and approved by Institutional Animal Care and Use Committee (IACUC) at Boston University Medical Campus.

## Author Contributions

KS, XW, and JH carried out the experiment. KS, MZ, and YZ analyzed the data. KS and JH wrote the manuscript. JH conceived the original idea and supervised the project. JH and MZ obtained the funding. All authors contributed to the article and approved the submitted version.

## Conflict of Interest

The authors declare that the research was conducted in the absence of any commercial or financial relationships that could be construed as a potential conflict of interest.

## Abbreviations

CVD: cardiovascular disease
ApoE^−/−^: ApoE deficient mice
GSH: glutathione
GSSG: oxidized glutathione
Grx1: glutaredoxin1
GR: glutathione reductase
GST-π: glutathione transferase pi.
